# Weighing serological evidence of human exposure to animal influenza viruses − a literature review

**DOI:** 10.2807/1560-7917.ES.2016.21.44.30388

**Published:** 2016-11-03

**Authors:** Reina Saapke Sikkema, Gudrun Stephanie Freidl, Erwin de Bruin, Marion Koopmans

**Affiliations:** 1 National Institute for Public Health and the Environment (RIVM), Centre for Infectious Diseases Research, Diagnostics and Screening (IDS), Bilthoven, the Netherlands; 2 Department of Viroscience, Erasmus MC, Rotterdam, the Netherlands

**Keywords:** influenza, zoonoses, zoonotic infections, avian influenza, emerging or re-emerging diseases, laboratory surveillance

## Abstract

Assessing influenza A virus strains circulating in animals and their potential to cross the species barrier and cause human infections is important to improve human influenza surveillance and preparedness. We reviewed studies describing serological evidence of human exposure to animal influenza viruses. Comparing serological data is difficult due to a lack of standardisation in study designs and in laboratory methods used in published reports. Therefore, we designed a scoring system to assess and weigh specificity of obtained serology results in the selected articles. Many studies report reliable evidence of antibodies to swine influenza viruses among persons occupationally exposed to pigs. Most avian influenza studies target H5, H7 and H9 subtypes and most serological evidence of human exposure to avian influenza viruses is reported for these subtypes. Avian influenza studies receiving a low grade in this review often reported higher seroprevalences in humans compared with studies with a high grade. Official surveillance systems mainly focus on avian H5 and H7 viruses. Swine influenza viruses and avian subtypes other than H5 and H7 (emphasising H9) should be additionally included in official surveillance systems. Surveillance efforts should also be directed towards understudied geographical areas, such as Africa and South America.

## Introduction

The family *Orthomyxoviridae* contains three distinct genera of influenza: A, B and C. Influenza A and B viruses are known to cause high human morbidity and mortality during the yearly seasonal epidemics. In contrast to influenza B viruses, influenza A viruses circulate in many animal species and are able to cross the species barrier*,* in particular from animal to human. This can occur either directly, or after a unique type of reassortment that results in the generation of viruses that are able to replicate in humans and have haemagglutinin (HA) genes (and less frequently neuraminidase (NA) genes) that are antigenetically distinct from those of seasonal influenza viruses (antigenic shift) [1]. Viruses resulting from ‘antigenic shift’ have caused four influenza pandemics in the past 100 years: the 'Spanish flu' A(H1N1) in 1918, the 'Asian flu' A(H2N2) in 1957, the 'Hong Kong flu' A(H3N2) in 1968, and most recently the A(H1N1)pdm09 pandemic in 2009 [1].

Animal influenza viruses are of concern because of the small but real risk of their adaptation to humans, possibly leading to efficient human-to-human transmission and sustainable circulation in the human population. It has been suggested that rising global trade and travel and changes in human demographics, consumption patterns and behaviours have caused an increase of emerging infectious diseases in general, and zoonotic influenza in particular [2-5]. Well-known examples of animal influenza viruses that have recently infected humans include A(H5N1), A(H6N1), A(H7N9), A(H9N2) and A(H10N8) [6].

To improve human influenza surveillance and preparedness, it is important to be able to assess influenza A virus strains circulating in the animal population as to their potential to cross the species barrier and cause human infections. The first step is to collect and review existing scientific studies that assess the prevalence of zoonotic influenza in human populations. Recently, a comprehensive literature review listed published virological evidence for human infection with swine and avian influenza viruses other than A(H5N1) [6].

While surveillance based on virologically-confirmed human influenza cases has a high positive predictive value, the approach has some downsides. Virus shedding in infected persons typically lasts only a week and has often diminished or ended by the time of sampling [7]. In addition, infections may cause only mild illness, leading to cases possibly remaining undetected. Studies investigating serological evidence of infection have a wider window of detection and have been used to study exposure in human–animal interface settings. A pitfall is that serological data need to be interpreted with caution due to cross-reactivity of antibodies among and within virus subtypes and the problems of sensitivity and reliability of standard serological tests when used to detect antibodies against novel influenza subtypes [8-11].

In this review, we assess studies describing serological evidence of human infection with animal influenza viruses. A scoring system was developed to assess the specificity of the obtained serology results in the selected articles, taking into account both the study design and the laboratory method used. This scoring system was used to weigh the serological evidence for animal influenza exposure in humans. This review can serve as input for an evidence-based risk assessment framework to evaluate novel influenza viruses or variants in light of their potential to create human outbreaks.

## Methods

### Search strategy and selection criteria

We performed a comprehensive literature search for serological studies dealing with zoonotic influenza, using the same search strategy as described in Freidl et al. 2014, but expanding the search period up to February 2014 [6]. The total period covered was from 1946 to February 2014. We additionally conducted a more cursory search to include studies published between February and December 2014.

Two investigators first screened all recovered publications by title and, when necessary, by abstract. They selected reports presenting serological evidence from observational studies describing human infection with animal influenza viruses. Studies of influenza A(H5N1) were excluded, as serological evidence of H5N1 in humans has been extensively reviewed previously [12,13].

The selected studies excluded those describing influenza antibody findings only in animals and those reporting only human-to-human transmission of animal influenza viruses. We also disregarded reviews, commentaries, and articles describing data that were described in previous publications.

### Scoring the quality of the evidence

To be able to assess the value of the outcomes of the selected studies, we developed a scoring system (Table 1, Table 2). For this, we identified important parameters for the evaluation of the specificity of results from observational studies describing serological evidence of human exposure to animal influenza viruses (i.e. study design, laboratory method used, background data on vaccination, exposure data). Subsequently we defined subsets for each parameter, based on review of the literature and Consortium for the Standardization of Influenza Seroepidemiology (CONSISE) and World Health Organization (WHO) recommendations [14,15,16]. Finally we assigned arbitrary points for considered parameters (or subsets thereof) to allow each individual study to be assigned a score; the score of a study was obtained by summing up the points corresponding to the parameters considered in that study. The final scoring system was discussed with virologists and epidemiologists from Erasmus University Medical Center (ErasmusMC) and Oxford University Clinical Research Unit (OUCRU) in Vietnam.

**Table 1:** Scoring system for evaluation of published reports describing seroprevalence studies of zoonotic influenza virus infections

Parameter	Maximum score	Individual scores			
		0	1	2	3
Control group	6	No	Unmatched	Age-matched (2)^a^ Sex-matched (2)^a^ Area-matched (2)^a^	NA
Repeated sampling^b^	2	No	NA	Yes	NA
Correction for age or reporting of study participants’ age groups^c^	1	No	Yes	NA	NA
Human vaccination status reported	1	No	Yes	NA	NA
Testing included human influenza type(s)	1	No	Yes	NA	NA
Other evidence	3	No	Serological evidence in animals to which humans were exposed	Virological evidence^d^ in animals to which humans were exposed	Virological evidence^d^ in human study participants
Laboratory method	5 (Table 2)	NA	NA	NA	NA
Total	18	NA	NA	NA	NA

NA: not applicable.

^a^ Two points are added to the final scoring result for age-matched, sex-matched and area-matched controls (same country), adding up to a maximum score of six.

^b^ Sampling to assess changes in antibody levels.

^c^ Score applied only if there was no age-matched control group.

^d^ Virus detection by culture or (real-time) reverse transcription-polymerase chain reaction (rtRT-PCR) and sequencing is listed as virological evidence.

**Table 2:** Scores assigned to published studies on zoonotic influenza viruses, according to the initial screening laboratory method used to evidence zoonotic influenza, and the subsequent method for confirmation

Confirmation method	Screening method			
	NT^a^	HI	ELISA	None^b^
NT^a^	NA	5	5	3
HI	5	NA	4	2
ELISA	5	4	NA	2
Western blot	5	4	4	0
NI	3	3	3	0
None^b^	3	2	2	NA

ELISA: enzyme-linked immunosorbent assay; HI: haemagglutination inhibition assay; NA: not applicable; NI: neuraminidase inhibition assay; NT: neutralisation test.

^a^ Neutralisation test: microneutralisation assay or virus neutralisation assay.

^b^ No description of method provided.

The maximum score that a study could obtain was 18. A detailed breakdown of the scoring system is shown in Table 1. Based on their overall score, we assigned all studies into four categories (A, B, C, D), ranging from best to worst. Category A spanned studies with scores ranging from 15 to 18 points, category B from 10 to 14 points, category C from 5 to 9 points and category D from 0 to 4 points.

### Rationale of the scoring system

In our scoring system, studies including a control group matched for age (less than 10 years difference in average age), sex (less than 10% difference in the percentage of women and men) and area (same country) received a higher score (Table 1: 6 of 18), as age, sex and location are possible confounding factors for influenza serology [17-20]. The inclusion of an age-stratified control group is also recommended by the CONSISE [14]. A control group is of particular interest for zoonotic influenza serology, because influenza infections occur repeatedly over a human lifetime, boosting pre-existing antibodies against human influenza viruses which might cross-react with animal influenza virus subtypes [21-26]. Comparison of an animal-exposed study population with a well-defined non-animal-exposed control group is important to avoid over-estimation of the significance of the serological findings. We also assigned a higher score to studies that did not include an age-matched control group but did report the age of study participants and corrected their results for age differences, or to studies that stratified their findings in separate age groups.

An antibody titre rise between two samples from the same individual was considered a more reliable measure of infection than obtained by a single serum sample, as individuals served as their own control.

A higher score was assigned to studies which addressed the possibility that antibodies may result from cross-reactivity among influenza subtypes. These studies included vaccination rates and/or tested for human influenza types, both variables known to have an effect on the generation of cross-reactive antibodies [17,27]. In order to score all studies in an objective manner, we did not evaluate their analysis of cross-reactivity but assigned scores based only on their inclusion of vaccination rate and/or testing for any human influenza type.

A higher score was also assigned to studies that added non-serological evidence of exposure of humans, particularly when they provided virological evidence for infection with animal influenza in their human study participants or in the animal population to which the participants had been exposed.

The rationale for our scoring of the laboratory methods used in the studies that we reviewed is based on the official WHO case definitions for human infections with influenza A(H5N1) virus [15]. A confirmed case, according to WHO, has a fourfold or greater rise in neutralisation antibody titre or a microneutralisation (MN) antibody titre of 1:80 or greater and a positive result using a different serological assay for example a haemagglutination inhibition (HI) titre of 1:160 or greater or a specific Western blot positive result.

Therefore studies that used both HI and neutralisation assays received the highest scores possible [5] as well as neutralisation assays that were confirmed with Western blot or enzyme-linked immunosorbent assay (ELISA) tests (Table 2). Moreover, studies using a confirmation test scored higher than studies that did not include a second serological assay to confirm their results. A single neutralisation test received three points in part because the WHO considers the MN assay to be the recommended test for measuring antibodies against highly pathogenic avian influenza A viruses. The MN test is an assay with high specificity [28]. The HI test is also a reliable serological test for influenza antibodies, and studies using this assays therefore receive the second highest score. The NA inhibition (NI) test can play a role in confirmation of influenza A subtypes but is not sufficient as screening test or if used as the only serological assay.

### Analysis of the data

All statistical analyses were performed in STATA (StataSE 13.0). For all analyses, a p-value of less than 0.05 was considered statistically significant.

## Results

### Search output

The final output of the literature search was 94 articles [10,18,19,29-119] (Table 3). Some articles could describe more than one study design, animal species, or influenza A subtype. Included were 12 prospective cohort studies, 13 cross-sectional studies in the general population or in rural populations, 57 cross-sectional studies in populations with routine exposure to animals, nine cross-sectional studies in hospital populations, and 11 animal influenza outbreak investigations. We found one report on an investigation of human serological evidence for canine influenza, four studies for equine influenza, 39 for swine influenza, and 56 for avian influenza A. The majority of the studies investigated serological evidence for antibodies to avian subtypes with HA-type H9 (43 articles), H7 (40 articles), H5 (excluding H5N1, 27 articles), and swine influenza subtype H1N1variant (H1N1v) (36 articles) (Table 3). 

**Table 3:** Results of literature search on zoonotic influenza viruses, 1946–2014 (n=94 publications)

Influenza virus	Influenza subtype	Number of studies included^a^	Laboratory methods used^b^ (number of studies)	Number of studies detecting antibodies in study group (proportion of included studies)	Number of studies detecting significant difference with control group^c^ (studies detecting significant difference with control group/total number of studies with control group)
**Canine**	H3N8	1	MN/NT and NI (1)	1 (1/1)	0 (0/1)
**Equine**	H3N8	4	MN/NT and HI (2) MN/NT (2)	4 (4/4)	0 (0/2)
	H7N7	2	MN/NT and HI (2)	0 (0/2)	0 (0/0)
**Swine**	H1N1v	35	MN/NT and HI (3) HI and NI (6) HI (22) MN/NT (3) ELISA (1)	32 (32/35)	13 (13/20)
	H1N2v	6	MN/NT and HI (1) HI (5)	6 (6/6)	5 (5/6)
	H2N3v	1	MN/NT and HI (1)	1 (1/1)	0 (0/1)
	H3N2v	11	MN/NT and HI (2) HI and NI (1) HI (7) ELISA (1)	9 (9/11)	3 (3/7)
**Avian**	H1	4	MN/NT and HI (1) MN/NT (2) Single radial analysis (1)	1 (1/4)	0 (0/1)
	H2	5	MN/NT and HI (2) MN/NT (2) Single radial analysis (1)	2 (2/5)	0 (0/1)
	H3	5	MN/NT and HI (2) HI and NI (2) Single radial analysis (1)	3 (3/5)	0 (0/1)
	H4	22	MN/NT and HI (2) HI and NI (3) MN/NT (15) HI (1) Single radial analysis (1)	6 (6/22)	1 (1/14)
	H5 (not H5N1)	27	MN/NT and HI (5) MN/NT and Western blot (1) MN/NT (18) HI (2) Single radial analysis (1)	12 (12/27)	4 (4/16)
	H6	21	MN/NT and HI (3) MN/NT (15) HI (2) Single radial analysis (1)	12 (12/21)	2 (2/13)
	H7	40	MN/NT and HI (10) HI and NI (3) MN/NT (17) HI (9) Single radial analysis (1)	16 (16/40)	6 (6/23)
	H8	16	MN/NT and HI (2) HI and NI (1) MN/NT (12) Single radial analysis (1)	2 (2/16)	1 (1/11)
	H9	43	MN/NT and HI (10) MN/NT and Western blot (1) HI and ELISA (1) MN/NT (17) HI (13) Single radial analysis (1)	37 (36/43)	13 (13/29)
	H10	19	MN/NT and HI (3) MN/NT (13) HI (2) Single radial analysis (1)	6 (6/19)	1 (1/12)
	H11	19	MN/NT and HI (2) HI and NI (3) MN/NT (12) HI (1) Single radial analysis (1)	9 (9/19)	0 (0/11)
	H12	14	MN/NT and HI (2) HI and NI (1) MN/NT (9) HI (1) Single radial analysis (1)	5 (5/14)	0 (0/7)
	H13	4	MN and HI (2) HI (2)	1 (1/4)	0 (0/1)
	H14	1	MN and HI (1)	0 (0/1)	0 (0/1)
	H15	1	MN and HI (1)	0 (0/1)	0 (0/1)
	H16	1	MN and HI (1)	0 (0/1)	0 (0/1)

ELISA: enzyme-linked immunosorbent assay; HI: haemagglutination inhibition assay; MN: microneutralisation assay; NI: neuraminidase inhibition assay; NT: neutralisation test.

^a^ A given article could describe more than one study design, animal species, or influenza A subtype, so the total number of studies in this column is greater than 94.

^b^ More detailed information can be found in the supplementary table (http://www.erasmusmc.nl/viroscience/research/suppl-table-animal-influenza-human-serology.pdf/?view=active).

^c^ Studies in which a significant difference was explicitly mentioned or for which a significant difference could be calculated based on the data provided.

**Figure 1:**
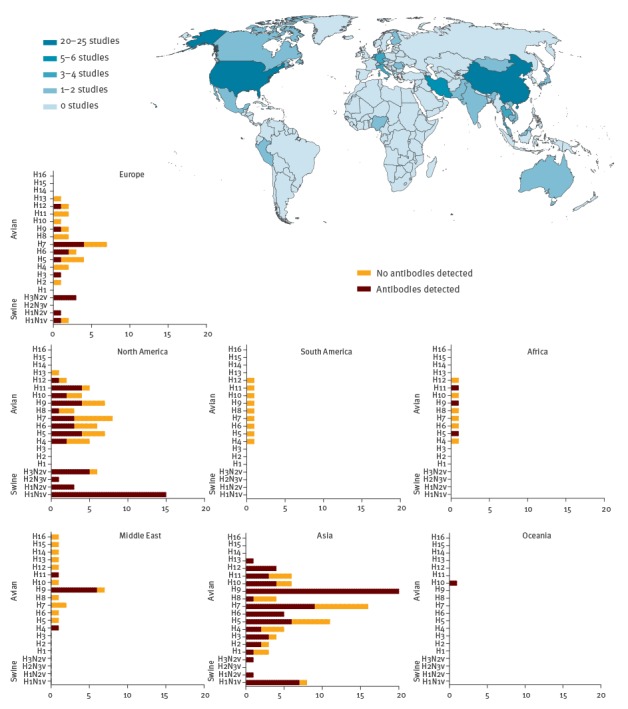
Geographical origin of animal influenza serological studies in humans, 1946–2014 (n=94 studies)

Study populations were from Asia (n = 37), North America (n = 28), Europe (n = 19), and the Middle East (n = 7, of which 5 were from Iran). For Africa, Oceania and South America, the search yielded only one publication for each. In North America, most studies focused on human infections with swine influenza, whereas in other parts of the world, such as Asia, more emphasis was placed on avian influenza viruses (Figure 1).

The X-axes of the charts represent the number of publications from that geographical area. The Y axes show the influenza subtypes.

**Figure 2:**
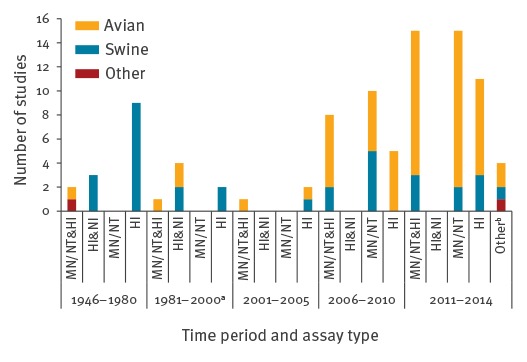
Diagnostic methods used in serological studies investigating animal influenza exposure of humans according to time period, 1946–2014 (n=94 studies)

HI: haemagglutination inhibition assay; MN: microneutralisation assay; NI: neuraminidase inhibition assay; NT: neutralisation test.

^a^ One study used single radial analysis.

^b^ One study used MN and Western blot as a diagnostic method, two used HI and enzyme-linked immunosorbent assay (ELISA) and one study used MN and NI assays.

In studies investigating swine influenza, the HI test was used more frequently, while studies that measured human antibodies against avian influenza viruses more often made use of neutralisation tests. Moreover, only studies published in the last decade used neutralisation as single diagnostic test. The use of NI assays on the other hand was only described in one of 72 included articles that were published after 2000, compared with seven of 22 studies that were published in the year 2000 or before (Figure 2).

Description of positive cases according to the WHO confirmed case definition could only be extracted from 11 articles [50,55,69,80,87,89,91,99,111,112,115]. These articles used the appropriate diagnostics tests and either reported the antibody titres or used the appropriate cut offs when describing the results.

### Scoring the studies

**Figure 3:**
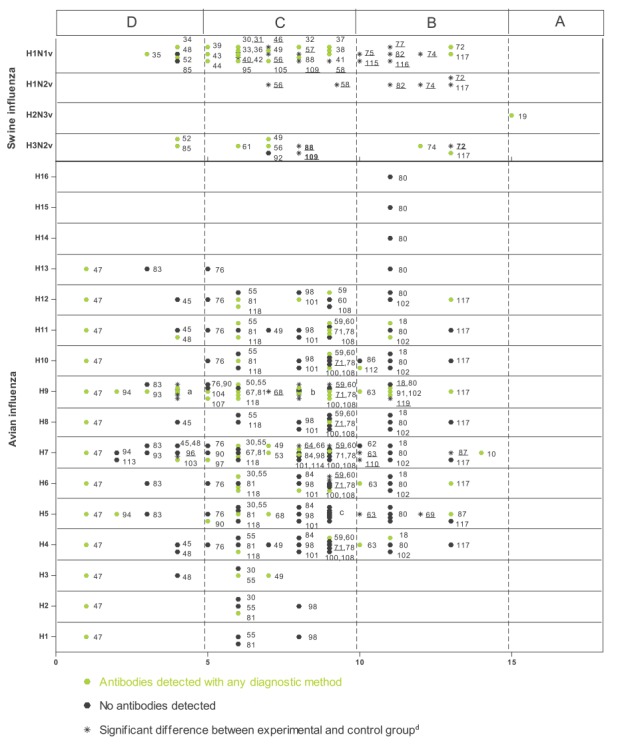
Scoring results of the included swine and avian influenza serological studies in humans, 1946–2014 (n=94 studies)

^a^ Reference numbers 51,54,70,73,79,96,103.

^b^ Reference numbers 64,66,84,89,98,99,101,111,114.

^c^ Reference numbers 59,60,65,71,78,100,108.

^d^ Reference numbers of studies with significant difference between experimental and control group are also underlined.

An overview of the scoring of all studies investigating serological evidence of swine and avian influenza viruses in humans is presented in Figures 2 and 3, respectively. Assuming an arbitrary quality threshold at 9 points (half the maximum score), only 24% of the studies (n = 23) were graded A or B, of which only one met the requirements for grade A. A total of 57% (n = 53) and 19% (n = 18) of the studies fell into category C and D, respectively. All but four grade A and B studies had a control group that was matched for at least two of the three desired characteristics (age, sex and area). Such controls were missing or insufficiently matched for most of the grade C studies and all of the grade D studies. The second marked difference was the fact that grade A and B studies more often included a serological confirmation test compared with C and D studies. Of the 23 studies graded A or B, eight investigated serological evidence for swine influenza (H1N1v, H1N2 variant (H1N2v), H2N3 variant (H2N3v), and H3N2variant (H3N2v)), 14 pertained to avian influenza viruses (H4–H16), and one study investigated human antibodies against a canine influenza virus (H3N8). Studies that fell into category A or B were all published relatively recently: category A or B swine influenza studies were all published in 2010 or later, and category A or B avian influenza studies were all published in or after 2006.

### Swine influenza

#### Cross-sectional studies

**Figure 4:**
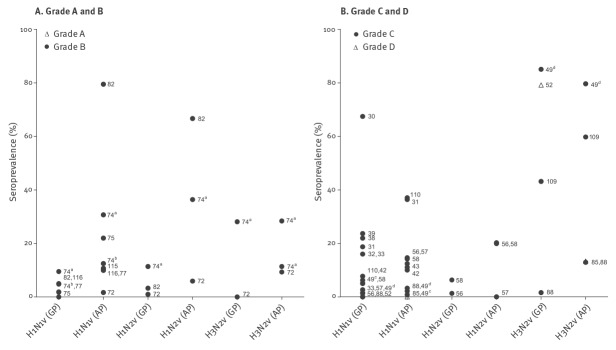
Seroprevalence of antibodies to different swine influenza viruses in exposed and control humans, 1946–2014 (n=39 studies)

GP: general population or control population; AP: animal-exposed population.

The vast majority of the swine influenza sero-epidemiological studies detected antibodies in a proportion of the population under investigation (32/35 of studies looking for H1N1v antibodies, 6/6 for H1N2v, and 9/11 for H3N2v) (Table 3). When single serum samples were analysed, cut-off values of serological assays (HI-assays and neutralisation assays) ranged from 1:10 to 1:100. For paired sera, a fourfold titre rise was considered proof of infection, but in cohort studies any titre increase during the study period was reported. The reported seroprevalences differed greatly among studies. In populations occupationally exposed to swine, the prevalence of antibodies to H1N1v ranged from 0% to almost 80% [82,85], to H1N2v from 4% to 67% [72,82] and to H3N2v from 9% to almost 80% [49,72]. Looking only at the high quality studies (grades A or B), the reported prevalences were similar to those for C and D studies, with the exception of H3N2v, for which the highest reported seroprevalence was 28% [74] (Figure 4). In the unexposed control groups the antibody prevalence to H1N1v ranged from 0% to 18.7% [31,88]. One study, published in 1968, found a seroprevalence to H1N1v of 67.4% in the general population, but this could be explained by cross-reacting antibodies against the 1918 pandemic influenza virus in the older population [30]. For H1N2v and H3N2v the prevalence in the general population and control groups ranged between 1.0% and 11.4% and between 0% and 85.0%, respectively [49,72,74].

For cohort studies, the seroprevalence at enrolment was included.

^a^ cH1N1.

^b^ trH1N1.

^c^ Sw/Ita/786/88.

^d^ Sw/BJ/47/91.

In studies that investigated the difference between an unexposed control group and the study population (i.e. study participants exposed to animals), a significantly higher number of seropositive people was found in the study population of 13 of 18 of the H1N1v studies, five of six of the H1N2v studies, and three of seven of the H3N2v studies.

#### Cohort studies

Woods et al. and Terebuh et al. found titre increases in antibodies against H1N1v of 0% to 8.5% per year in serum of farm workers and abattoir workers exposed to swine [43,74]. Gray et al. found that 25% of rural residents showed a fourfold increase in antibodies to H1N1v over a two year period from 2004 to 2006 [58]. Slightly lower rates were found for H1N2v, for which 5% (fourfold antibody titre increase, rural residents) and 8% (antibody titre rise, farm workers and abattoir workers) of study participants had evidence of exposure over two years [58,74]. In the period from 2008 to 2011, both Coman et al. and Gray et al. found a high percentage of seroconversions for H1N1v and H1N2v, which were most likely due to cross-reactions with influenza A(H1N1)pdm09 [117,118]. Both Coman et al. and Terebuh et al. investigated serological evidence of H3N2v exposure during a time-period of two years, concluding that the number of titre increases for different types of H3N2v in the swine-exposed group were not significantly higher than in control groups [74,117].

#### Outbreak studies

The five outbreak studies included in this review targeted people who were exposed to swine infected with swH1N1 (n = 4) or swH2N3 (n = 1). Those that investigated people exposed to swH1N1 reported seroprevalences ranging from 15% to 40% (using various cut-offs), and three of four reported a significant difference between the exposed individuals and a control group [40,44,46,75]. All four H1N1v studies were graded C, except the study by DaWood et al., which received grade B. It reported that 40% of pig-exposed study participants had a MN titre ≥ 80 and HI titre ≥ 20 [75]. The one study of H2N3v, graded A, investigated workers who were exposed to H2N3-infected swine and found, remarkably, a higher seroprevalence in the unexposed control group [19].

### Avian influenza

#### Cross-sectional studies

**Figure 5:**
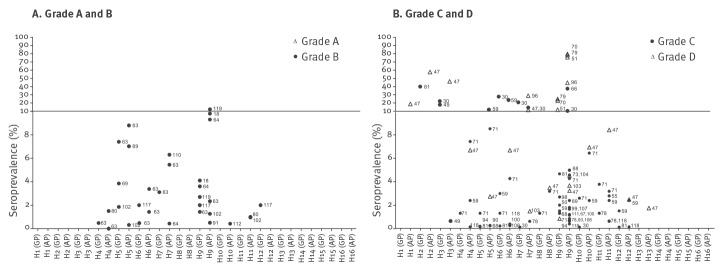
Seroprevalence of antibodies to different avian influenza viruses in exposed and control humans 1946–2014 (n=56 studies)

GP: general population or control population; AP: animal-exposed population.

Most studies screening for antibodies against avian influenza viruses failed to detect antibodies in any proportion of the population under investigation, except for subtype H9 to which 36 of 43 studies detected antibodies. The cut-off values for neutralisation tests used in the studies ranged from 1:2 to 1:160 for HI and 1:10 to 1:80. In the cross-sectional studies over all, antibodies were detected against avian influenza A virus HA subtypes H1 through H13. However, in studies graded A or B, fewer subtypes were detected: H5, H6, H7, H9, H10, H11 and H12 (Figure 5).

For cohort studies, the seroprevalence at enrolment was included.

When reviewing only studies that compared prevalence of antibodies in risk groups (subjects in contact with animals) with those from a control group, some studies found significant differences in seroprevalence between both groups for avian influenza subtypes H4 (1 of 14 studies; 1/14), H5 (4/16), H6 (2/13), H7 (6/23), H8 (1/11), H9 (13/29), and H10 (1/12). Insufficient standardisation or description of methods and cut-offs did not allow a direct comparison of the data.

Studies with a lower score (C or D) appeared to report higher prevalences for avian influenza antibodies than did A or B studies, but the difference was not significant (p > 0.05). Our conclusions were similar when we compared different cut-offs.

#### Cross-sectional studies: grade A and B studies

Most grade A and grade B studies reported serological evidence of H5, H7 and H9 exposure, but with considerable variation. Gray et al. found a significant difference in the seroprevalence of H5N2 antibodies in a swine- and poultry-exposed rural population from Iowa, United States, vs unexposed controls from the same region (8.8 vs 0%) [63]. Okoye et al. found H5N2 antibodies in poultry-exposed and -unexposed groups from Nigeria (0.3 vs 1.8%), however, this difference was not significant [63,102]. Moreover, two studies executed in Romania and Vietnam found no antibodies in either group [18,91]. Gray et al. also found neutralising antibodies to H6N2 and H7N2 influenza virus in the same study, but the prevalences were not significantly different between the exposed group and non-exposed controls (1.4 vs 4.0% and 5.5 vs 0%) [63]. Five other studies failed to find serological evidence of H6 or H7 exposure [18,63,80,102,117]. Only one-cross sectional study looked at H7N9 exposure, finding a seroprevalence of 6.3% in poultry workers in Guangdong, China, and a significantly lower percentage in non-exposed controls (0%) [110]. Antibodies to H9N2 avian influenza virus were found in four of seven studies, with seroprevalences ranging from 1.3 to 12.3% [91,119]. Only Wang et al. found a significantly higher prevalence in exposed vs control persons in Shanghai, China (5.0 vs 1.3%). Uyeki et al. included other H9 antigens and found a low level of antibodies to H9N3 and H9N7 virus in Vietnamese poultry workers, although the prevalences of 0.5% and 2.5%, respectively, were not significantly different from the control group (0 and 3.5%) [91]. In 2013, Qi et al. found 0.4% prevalence of antibodies to H10N8 in animal workers in Guangdong province, China, which did not differ significantly from the non-exposed controls (0%) [112].

#### Cohort studies

A two-year study from Gray et al. in Iowa found 0.6% of swine and poultry unexposed agricultural workers experiencing an antibody increase for H4N8 during this time period, and 0.8% of exposed and unexposed agricultural workers experiencing an antibody increase for H5N2 [63]. Four cohort studies found antibodies to H6N1 during their two-year study periods. The percentage of the study populations that experienced an increase in antibody titres ranged between 0.1% and 2% [101,108,117,118]. Two of the four studies could not find an association with animal exposure. A very low percentage (< 0.3%) of four of the study populations experienced a slight increase in antibodies against H4N6, H7N7, H10N4 or H12N5 [82,101,108,117].

Increases in H9N2 antibodies were detected in three two-year cohort studies investigating poultry workers. In Thailand, between 2008 and 2010, 2% of the study population seroconverted in the first year of the study, and 2.5% seroconverted in the second year [101]. A similar number of antibody titre increases was found in Mongolia between 2009 and 2011: 2.2% of the adults experienced an increase in antibodies after either year 1 or year 2 of the study [108]. In both studies there were individuals that showed a fourfold antibody titre increase (0.3% and 0.8%) but did not report influenza-like illness, suggesting subclinical infections [101,108]. Gray et al. found that 0.3% of agricultural workers from Iowa experienced a titre increase for H9N2 during the two-year study period [63].

Besides information about the number of seroconversions, cohort studies provide information on antibody longevity. Lu et al. show that all individuals previously seropositive for H7N7 and H9N2 sera became seronegative after one year [64]. Krueger et al. likewise found that antibody titres against H6N1 and H7N7 were undetectable after one year [101].

#### Outbreak investigations

The outbreak studies included in this review investigated people who had been exposed to poultry infected with H5, H7, H9 or H10. A study of H5N2 outbreaks at Japanese chicken farms found a positive H5N2 neutralising titre (MN ≥ 1:40) in 25% of the workers, of whom 7.8% showed a fourfold antibody increase [65]. Di Trani et al. found that 2.1% of Italian poultry workers exposed to H5N2- or H5N7-infected poultry showed an antibody titre (HI ≥ 1:10), but results were not confirmed by MN, nor was there a significant difference between study participants and unexposed controls [87].

The outbreak studies that found H7 antibodies reported seroprevalences from 0.4 to 3.2% in exposed poultry workers [53,87]. The outbreak study by Di Trani et al. found significantly more antibodies to H7N1 and H7N3 in H7-exposed poultry workers compared with unexposed controls (3.2 vs 0.8%) [87]. Another outbreak investigation conducted in Italian poultry workers likewise found H7N1 and H7N3 antibodies, in 0.4% and 2.2% of the workers respectively, but included no controls. Moreover, six of the 983 workers in that study reported conjunctivitis but showed no H7 antibody response [53]. Using an MN assay with cut-off of 1:80, Skowronski et al. found no evidence for human antibody responses in Canadian workers involved in an H7N3 outbreak in poultry; but they reported that close contact with the infected poultry correlated with red or watery eyes [62]. In a study of H7N7 by Meijer et al. in the Netherlands, the results from the HI assay (≥ 1:10) indicated a prevalence of 49%, but none of the titre rises could be confirmed by MN; however, ocular symptoms of infection appeared more frequently in subjects with HI-detected antibodies compared with subjects without antibodies [10].

One outbreak study investigating H9-exposed poultry farmers found antibodies in 11 of 34 participants (32.3%), but did not include a control group or describe a cut-off for the HI assay [54]. Arzey et al. investigated abattoir workers exposed to H10N7-infected poultry and found that two of seven reporting conjunctivitis were polymerase chain reaction (PCR)-positive for influenza A; partial sequence analysis of the HA confirmed the presence of H10 subtype, but the findings could not be serologically confirmed [86].

#### Equine and canine influenza

Two studies executed before 1970 in Europe investigated human exposure to equine influenza viruses and found prevalences from 4.2% to 20.9% for H3N8, using HI and neutralisation assays, but no non-exposed control group was included [29,30]. Khurelbaatar et al. also investigated exposure to equine influenza virus in a Mongolian rural population during a period from 2009 to 2011 and found a seroprevalence of 1.1% at enrolment. During the two-year follow-up period, 2.5% of the study population experienced a fourfold titre increase against equine influenza virus H3N8, but exposure to camels or horses was not associated with titres to H3N8 [100,108]. Antibody responses have been detected against canine influenza A(H3N8) in dog-exposed subjects, but comparison with an unexposed control group yielded no significant difference (20.7 vs 12.1%) [106].

## Discussion

There is currently no methodology or tool available for the quality assessment and comparison of influenza serology population studies [120]. In this review we therefore tried to develop a grading system to weigh the evidence for human infection with animal influenza viruses from the included studies. Each attribute of the grading system is either a known confounding factor, and should therefore be included in the analysis or is an accepted method to improve the specificity of the outcome of serological influenza. Although the weights of variables of the scoring system were divided in an arbitrary manner, the scoring system comprises important factors that should be incorporated in future studies investigating human exposure by animal influenza viruses to improve reliability of human serological evidence.

It was often not possible to score all aspects of the execution of a study, as details were lacking from its methods section. For example, although we scored for the presence of information on confounding factors, we did not take into account how they were incorporated in the analysis of the data because the methodological information was insufficient to allow this. Nor did we assess the quality and execution of the laboratory tests, because descriptions often omitted details that can very much influence test outcome, e.g. the origin and quality of red blood cells used in HI assays [121].

It is difficult to interpret and compare the diverse antibody titres reported in the literature. Several studies addressing the inter-laboratory variability of influenza HI and MN assays have found significant differences in geometric mean [8,121-124]. The interpretation of the test results is even more difficult because little is known about the agreement between HI and MN assays [122,124]. In addition, pre-existing antibodies against human influenza viruses may cross-react with animal influenza virus subtypes, resulting in titres that are unrelated to exposure or infection with an animal influenza virus [21-23]. Moreover, for many zoonotic influenza A subtypes the optimal detection method is unknown.

In this review, the focus was on assessing the specificity of the reported findings. However, it is possible that clinical or subclinical infections are being missed and that the actual rate of infection is higher than the serological data suggest. For example, individuals exposed to H7 (other than H7N9) have developed a virologically confirmed conjunctivitis when no seroconversion could be detected [10,62]. Also, infections with avian influenza causing fever and/or respiratory symptoms can sometimes be confirmed virologically but not serologically [10,110]. Moreover, serological responses to zoonotic influenza can wane rapidly, which can lead to underestimation of the frequency of spill-over of animal influenza viruses to humans [64,125].

Different levels of exposure also affect the level of human antibody titres against animal influenza viruses. Some studies compared occupational groups, and many of them find differences in seroprevalence between different occupational groups, which are postulated to reflect differences in exposure [31,42,43,49,56,62,67,68,70-73,79,93,96,116,117]. A problem is that influenza infections were rarely measured in the animals to which the study population was exposed at the time of the study, therefore making it difficult to assess the true levels of exposures. The lack of this information may in part explain the seemingly contrasting conclusions reached regarding the occupational groups that have the highest seroprevalence to animal influenza: some studies find the highest antibody titres in veterinarians [43,72] while others find the highest titres in farmers [56,70], abattoir workers [31] or poultry market workers [68]. Moreover, the term ‘occupational exposure’, as well as different occupational groups are loosely defined, which makes them very hard to compare. Ahad et al. for example found high titres of avian influenza antibodies in poultry vaccinators, but very few other studies have looked at poultry vaccinators as a separate group occupationally exposed to poultry [96].

Looking at the studies collected for this review, it becomes clear there is no agreement on the diagnostic methods, cut-offs or study design that should be used to investigate the prevalence of zoonotic influenza in humans. This limits both the interpretation and the comparability of the available data. Following the H1N1 pandemic in 2009, the WHO reached the same conclusions in a review on the pandemic and requested standardised methods to improve the comparability of the serological data [125]. Although the CONSISE published recommendations and protocols to standardise serological studies on zoonotic influenza virus outbreaks, human influenza virus epidemics, and seasonal influenza, there are no guidelines for the design and execution of population studies for influenza on the human–animal interface [126,127].

An interesting finding is that studies graded C or D in this review generally reported higher seroprevalences to avian influenza viruses in humans than A or B studies. It is possible that using a less stringent study protocol leads to an overestimation of serological findings of animal influenza in humans. Therefore, to increase the reliability of the evidence and reduce the occurrence of false positive outcomes, inclusion of confounding factors either in the study design or the data analysis is important.

In this review we see that antibodies to swine influenza viruses A(H1N1)v, A(H1N2)v, and A(H3N2)v are more prevalent among persons occupationally exposed to pigs compared with those not exposed. However, given the potential for cross-reactivity within subtype, serological studies that investigate human infection with swine influenza should be interpreted with great caution. Unlike avian influenza viruses, endemic swine influenza viruses often have common origins with seasonal human influenza viruses. Novel pandemic influenza human viruses have originated from swine viruses or have been introduced in swine, and play an important role in the evolution of genetic diversity of swine influenza viruses [128]. For instance, with the recent emergence of pandemic (H1N1) 2009 virus and subsequent reintroductions in the swine population, serological population studies investigating human infection with swine influenza viruses should be designed and interpreted with extreme caution to differentiate true exposures from cross-reactions [129,130]. Nevertheless, the number of studies finding a significant difference in seroprevalence between swine-exposed study groups and unexposed control groups is strong evidence of frequent spill-over events from swine to humans. The high number of reported swine-to-human transmissions and evidence for subsequent human-to-human transmission, in combination with the recent emergence of pandemic (H1N1) 2009 virus, warrants increased serological and virological surveillance of swine and people that are exposed to swine [6,131]. Unfortunately swine influenza surveillance is less prevalent than avian influenza surveillance, and there is relatively little knowledge on prevalence and circulation of swine influenza [132].

According to our assessment, the most reliable serological evidence (grade A and B) was found for human exposure to avian influenza virus HA-types H5, H7, and H9. The risk of infection with H5 and H7 subtypes is illustrated by the serious recent outbreaks of subtypes (H5N1) virus and A(H7N9) virus in humans, which, as of late 2016, have resulted in 452 and 320 deaths, respectively [133]. Few cases of humans infected with avian H9 viruses have been reported: Freidl et al. described in their literature review the virological evidence of 15 cases of humans infected with H9N2 [6]. However, avian H9N2 viruses are a growing concern, and the mild disease associated with H9 infection potentially leads to considerable underestimation of incidence [50,134,135]. H9 avian influenza can be found in poultry all over the world and is also described in multiple other avian species, pigs and dogs [136-139]. Moreover, internal genes of A(H9N2) were found in A(H10N8), A(H7N9) and A(H5N1), showing that A(H9N2) can reassort with other influenza subtypes, potentially resulting in the generation of new zoonotic influenza types [140-142]. In 2013, human cases of infection with H10N8 and H6N1 were reported, as well as presence of these serotypes in environmental samples from animal markets, showing that H6 and H10 serotypes can likewise pose a risk to human health [140,143-145]. Serological cohort studies of persons exposed to poultry provide information on the incidence and longevity of antibodies to zoonotic influenza viruses. For most avian influenza subtypes, this information is currently unknown and will greatly contribute to the risk analysis of zoonotic avian influenza.

Although human antibodies have been found against equine and canine influenza, these infections seem to be a minor public health risk. However, the very limited number of studies could lead to substantial under-reporting.

## Conclusion

Comparing human serological data is difficult due to a lack of standardisation in the collection of epidemiological data and the laboratory methods used in published zoonotic influenza studies. Researchers should take into account WHO guidelines, known confounding factors and the need for a control group in order to produce research articles that can be used and compared by policymakers and other researchers to better assess the risks and prevalence of animal influenza exposure in humans.

Swine-to-human transmission is prevalent, but national surveillance systems and standard serological surveillance of swine and human risk groups is scarce. Surveillance for avian influenza is more common, but most veterinary surveillance systems target H5 andH7 serotypes and, accordingly, most serological evidence is reported for these subtypes. Given the zoonotic potential of avian influenza viruses, which can potentially reassort with circulating seasonal human influenza virus subtypes, systematic surveillance in poultry populations should be expanded beyond H5 and H7, the primary focus for the veterinary sector [146,147]. Subtypes H6, H9 and H10 are known to be able to infect humans and should therefore be included. Moreover, structured surveillance of human risk groups to detect spill over of influenza viruses is rare and should be implemented in national surveillance systems. Finally, we found that the majority of studies conducted at the human–animal interface represent Asia, Europe, and North America. Efforts should be made to shed light on understudied areas, such as South America and Africa.
